# Contemporary Speculations and Insightful Thoughts on Buckwheat—A Functional Pseudocereal as a Smart Biologically Active Supplement

**DOI:** 10.3390/foods13162491

**Published:** 2024-08-08

**Authors:** Vladimir S. Kurćubić, Slaviša B. Stajić, Vladimir Jakovljević, Vladimir Živković, Nikola Stanišić, Pavle Z. Mašković, Vesna Matejić, Luka V. Kurćubić

**Affiliations:** 1 Department of Food Technology, Faculty of Agronomy, University of Kragujevac, Cara Dušana 34, 32102 Čačak, Serbia; 2 Faculty of Agriculture, University of Belgrade, Nemanjina 6, 11080 Belgrade, Serbia; stajic@agrif.bg.ac.rs; 3 Department of Physiology, Faculty of Medical Sciences, University of Kragujevac, 69 Svetozara Markovica St., 34000 Kragujevac, Serbia; drvladakgbg@yahoo.com (V.J.); vladimirziv@gmail.com (V.Ž.); 4 Department of Human Pathology, Sechenov First Moscow State Medical University, 8 Trubetskaya St., 119991 Moscow, Russia; 5 Institute for Animal Husbandry, Belgrade-Zemun, Highway to Zagreb 16, 11000 Belgrade, Serbia; nikola0135@yahoo.com; 6 Department of Chemistry and Chemical Engineering, Faculty of Agronomy, University of Kragujevac, Cara Dušana 34, 32000 Čačak, Serbia; pavlem@kg.ac.rs; 7 Department of Medical Microbiology, University Clinical Center of Serbia, Pasterova 2, 11000 Belgrade, Serbia; kurcubiclk@gmail.com

**Keywords:** buckwheat, nutritional value, bioactive compound (BAC), phytochemicals, flavonoids, gluten-free, antimicrobials, antioxidants, healthier food, functional food

## Abstract

Today, food scientists are interested in more rational use of crops that possess desirable nutritional properties, and buckwheat is one of the functional pseudocereals that represents a rich source of bioactive compounds (BACs) and nutrients, phytochemicals, antimicrobial (AM) agents and antioxidants (AOs), which can be effectively applied in the prevention of malnutrition and celiac disease and treatment of various important health problems. There is ample evidence of the high potential of buckwheat consumption in various forms (food, dietary supplements, home remedies or alone, or in synergy with pharmaceutical drugs) with concrete benefits for human health. Contamination as well as other side-effects of all the aforementioned forms for application in different ways in humans must be seriously considered. This review paper presents an overview of the most important recent research related to buckwheat bioactive compounds (BACs), highlighting their various functions and proven positive effects on human health.

## 1. Introduction

Buckwheat, especially common (*Fagopyrum esculentum*) and Tartary buckwheat (*Fagopyrum tartaricum*), has attracted research interest from food scientists due to its high efficiency as a functional food. From a nutritional point of view, buckwheat is one of the most nutritious foods, with the most complete composition, because its grains are rich in proteins with a balanced composition of amino acids, gluten-free flour, dietary fibre, vitamins, resistant starch, phytosterols, fagopyrins, fagopyritols, d-fagomine, flavonoids, and phenolic compounds/phenolic acids, and they have demonstrated beneficial properties in the treatment of chronic diseases [1,2,3,4].

Buckwheat is a significant source of bioactive compounds (BACs) that have health and nutritional importance [2,3]. The nutritional composition of buckwheat is shown in Table 1. The high content of vitamins and minerals in buckwheat with an extremely high level of phenolic compounds and confirmed positive effects on human health makes it a very potent food for incorporation into healthier or functional food after various types of processing [4,5].

**Figure 1:**
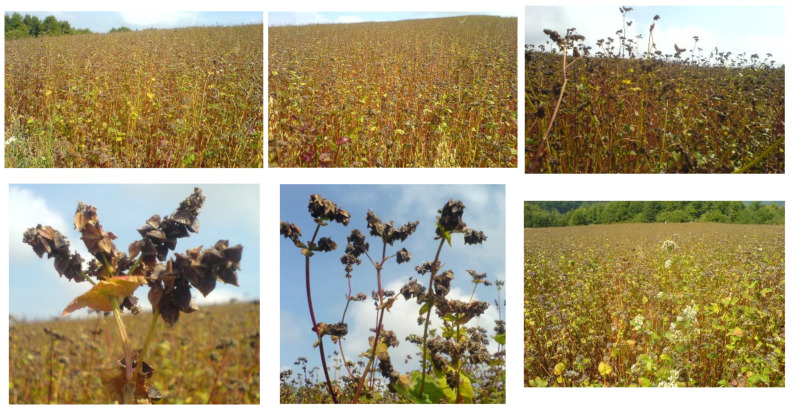
Experimental fields with *Fagopyrum esculentum* (Kopaonik Mountain, Serbia), maturity stage.

**Figure 2:**
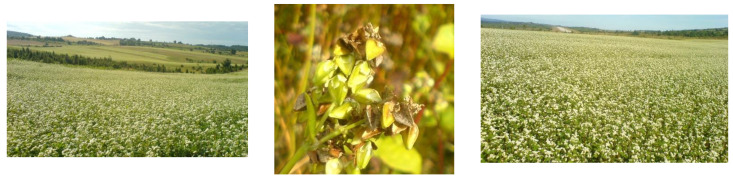
Experimental fields with *Fagopyrum esculentum* (Ponikve, Tara Mountain, Serbia), flowering phase.

The growing popularity of buckwheat in the diet is explained by its outstanding nutritional quality and technological properties. Nowadays, the consumption of buckwheat is not aimed at eradicating hunger, but at enriching the gastronomic offer. Consumers appreciate its favourable nutritional composition, healthfulness, and interesting taste, as well as the ever-increasing range of products and innovative buckwheat dishes: buckwheat products without gluten, sugar, and lactose, as well as buckwheat products with less salt, as part of the healthy food offer [6,7]. Experimental fields with *Fagopyrum esculentum* are shown in Figure 1 (maturity stage) and Figure 2 (flowering phase).

Pseudocereals, as one of any of the non-grasses that are used in much the same way as cereals, are described as “the grains of the twenty-first century” due to their excellent nutritional value. They are rich in starch, fibre, proteins of high quality with a balanced essential amino acid composition, minerals (calcium, iron and zinc), vitamins, and phytochemicals such as saponins, polyphenols, phytosterols, and phytosteroids [8]. Tartary buckwheat contains more valuable nutrients than common buckwheat, but it also contains allergenic proteins that can cause allergic reactions through an IgE-mediated response with clinical symptoms such as eczema, asthma, dermatitis, and anaphylactic shock. Similarly, buckwheat has been identified as one of the major food allergens, resulting in severe and dangerous symptoms when ingested or inhaled in small amounts, such as in flour and related products [9,10]. The fermentation process has the potential to reduce sensitization to buckwheat, but it is important to note that fermentation can also create new allergen epitopes, which could increase sensitization to buckwheat. Therefore, it is important to be careful and avoid over-fermentation, because excessively striving for low allergenicity can lead to the loss of the original nutritional value of Tartary buckwheat or the development of unfavourable flavours [11]. Both species present excellent dietary sources of phenolic compounds, rutin (RUT) and quercetin (QE). In Tartary sprouts, RUT represented 90% of the total content of phenolics, and only 20% in common sprouts [5].

The main molecules in buckwheat that have biological activity associated with positive effects on human health are RUT and QE, both of which are more abundant in *F. tataricum* than in *F. esculentum* [5]. The predominant function of RUT is to prevent the occurrence of oxidative stress and inflammatory responses by removing ROS or preventing their formation [12], directly and positively correlating with its antioxidative (AO) activity. Combined with ascorbic acid, AOs act as anti-inflammatory and anti-apoptotic agents against skin damage caused by UV radiation, indicating that both substances potentially exert a cytoprotective effect [13]. The authors report that RUT has significant potential in the fight against neurodegenerative diseases by reducing pro-inflammatory cytokines and improving the activity of AO enzymes [14]. The above-mentioned activity of AOs is closely related to their anticancer properties, inhibition of proliferation, attenuation of superoxide production and reduction of adhesion and migration of human cancer cells [15].

Sofi et al. [3], in their updated review, comprehensively and thoroughly described the nutritional and bioactive characteristics of buckwheat as well as its potential for designing and creating gluten-free products. In chapter 5, the authors succinctly described a multitude of health-promoting attributes of buckwheat: AO, antiallergic, anticancer, hepatoprotective, antidiabetic, antihypertensive, antihyperlipidemia, and antineurodegenerative activities, anti-inflammatory and antifatigue effects, and antigenotoxicity [3].

## 2. Bioactivity, Bioavailability, and Absorption of Buckwheat

### 2.1. Phytochemical Profiles of Buckwheat

Why did we choose buckwheat for speculation out of 1340 plants proven to contain approximately 30,000 BACs [16]?

This plant has attracted research interest due to its high AO content and is associated with various health benefits, including improved heart health, better blood sugar control and digestive health support, Table 1 [17].

Several BACs have been identified in different parts of the plant (leaves, seeds, roots, etc.), in various buckwheat species. These compounds include flavonoids, phenolic acids and their derivatives, tannins, fagopyrin, triterpenoids, steroids, stilbenes, and so on [17].

The major phenolic compounds in Tartary buckwheat flour are flavonoids (6.65–22.74 mg/g, dry weight (DW)) [18,19]. Different types of flavonoids have been identified in buckwheat root, flower, fruit, seed, germinated seed, seedling, seed coat and processed foods [20]. The content of flavonoids depends on various factors, including the growth stage of the plant, the organ, the type of buckwheat grown, the growing season and the growing region [21,22].

Various flavonoid subfamilies have been identified in Tartary buckwheat by different detection methods, e.g., high performance liquid chromatography (HPLC), HPLC coupled with mass spectrometry (HPLC-MS), ultra-HPLC coupled with MS by electrospray ionization (UPLC-ESI-MS) and nuclear magnetic resonance (NMR), and subdivided into flavonols, flavones, isoflavones, flavanones, flavanols/flavan-3-ols, anthocyanins, fagopyrins, proanthocyanidins and flavonolignans [17].

Buckwheat flavonoids can scavenge free radicals and prevent various diseases such as cancer, cardiovascular disease, and cerebrovascular and degenerative diseases [23].

The importance of using buckwheat in the diet is reflected in the fact that it contains the largest amount of vegetable proteins, but also due to the intake of a certain amounts of quercetin, which is most abundant in buckwheat and rose hip (*Rosa canina*).

Among flavonoids, RUT (6.06–18.67 mg/g) and QE (0.31–2.38 mg/g) are present in the highest amounts in Tartary buckwheat flour [18,19]. RUT is the main flavonol in buckwheat, comprising 90% of the total phenolics and is widely distributed in plants but is rarely present in their edible parts. Except in buckwheat, this flavonoid was not detected in other cereals and pseudocereals. In addition to the grain, the other above-ground parts of Tartary buckwheat also contain RUT [24,25]. The content of RUT is higher in buckwheat flowers (47–63 mg/g), than in stems (6–14 mg/g) and roots (3–8 mg/g) [19]. RUT, one of the most well-known AOs found in buckwheat, possesses anti-inflammatory and anti-cancer properties. It also protects blood vessels, helps strengthen capillaries and reduces the risk of cardiovascular diseases [22]. Buckwheat (*Fagopirum* spp.) crop production at the world and national levels is presented in Table 2, from the most extensive production to the smallest, in descending order. Bioactivities and BACs are detailed in Table 3.

Actually, RUT is a QE glycoside, and in addition to the structural similarity, these compounds exhibit similar biological properties. After mixing ground buckwheat products with water, RUT is converted to the flavonoid QE by the hydrolysis enzyme rutinosidase [43,52].

In addition to RUT and QE, numerous other flavonols have been isolated in buckwheat, such as kaempferol, myricetin, isoquercetin, etc. Among the flavones, another subgroup of flavonoids, orientin, vitexin, homoorientin and isovitexin were detected in buckwheat seeds. Additionally, hesperetin, naringenin, phloretin and other BACs were isolated from flavanones, as well as catechin and epicatechin derivatives from flavanols [17,21].

In addition to flavonoids, buckwheat is the richest source of phenolic acids, which also contribute to AO properties [54]. Phenolic acids, as natural AOs, act against reactive oxygen species and reduce cardiovascular diseases, cancer, and aging [55]. Some of the common phenolic acids found in buckwheat include chlorogenic, caffeic, ferulic, gallic, p-hydroxybenzoic, protocatechuic, syringic, p-coumaric, and vanillic acids [54].

It has been reported that the content of phenolics and antioxidant capacity are significantly higher in the flour of buckwheat compared with selected gluten-free flours from cereals, pseudo-cereals and legumes, suggesting that buckwheat could be an excellent source of phenolic compounds, Table 4 [76]. The in vitro antioxidant capacity was evaluated in terms of both ferric reducing antioxidant power (FRAP) and oxygen radical antioxidant capacity (ORAC), expressing results as gallic acid equivalent (GAE) and trolox equivalent (TE) [76].

### 2.2. Mechanisms of Action of Buckwheat BACs

The high content of RUT and QE in Tartary buckwheat and the high AO activity have further implications for cytotoxic and antigenotoxic experiments in vitro and on animal models. A study on the antigenotoxic activity of Tartary buckwheat in human hepatoma cell lines has shown that flavonoid components in Tartary buckwheat products are more effective in maintaining health in their complexed form than the individual active ingredients, RUT or QE.

Based on experiments in cell lines and animal models of breast, colon, skin and other cancers, it is hypothesized that Tartary buckwheat metabolites RUT and QE may be effective against mammary, colon, skin, and other cancers [65]. Moreover, RUT and QE exhibit hepatoprotective, antibacterial, antiviral, antiulcer, cardioprotective, antithrombotic and many other biological properties [17].

Recent research has identified potential health benefits of food proteins and bioactive peptides, with the already proven desirable activities of polyphenols [77]. AO, AM, antihypertensive and antidiabetic activity has been reported in various studies, meaning that common buckwheat is becoming an important food ingredient that should prevent, and/or be used to treat patients suffering from, various chronic diseases [78]. Buckwheat flavonoid’s structure is interesting because of an active phenolic hydroxyl group with the ability to scavenge free radicals and that may have a number of beneficial bioactivities in the prevention of cancer, cardiovascular disorders, aging, and cerebrovascular and degenerative diseases [23,54].

Flavonoids play an important role in humans and plants, so QE is one of the most commonly commercially available natural BACs that have an advantage over conventional drugs because they have fewer side effects. Due to the low solubility and poor bioavailability of QE, its encapsulation in macromolecules increases its bioavailability and pharmaceutical efficiency [50].

The AO effects of Tartary buckwheat bran extract (TBBE) on the lipid profile were investigated in hyperlipemic rats fed a high-fat diet. In those experimental rats, TBBE was proven to effectively reduce serum total triglycerides (TGs) and total cholesterol (TC) compared to control groups (*P* < 0.05), as well as liver TC and TGs by 36.4 and 73.9% in the low-dose group compared with the high-fat group (*P* < 0.05). Such effects are considered outstanding in reducing serum triglycerides, anti-atherosclerosis and resistance to serum-lipid oxidation. TBBE increased both serum glutathione peroxidase (GSH-Pk) activity and the antiatheromatous plaque formation index (AAI). Consequently, the plasma atherogenic index (AIP), atherogenic index (AI), and serum malondialdehyde (MDA) were decreased compared to control groups (*P* < 0.05 or *P* < 0.01). TBBE significantly reduces TGs and TC in the serum and liver of rats, increases the AO effects in the serum, and inhibits the formation of serum lipid peroxide [79].

In view of all of the different mechanisms of action and applications in different areas described above, in future, research should focus on ensuring the development of effective strategies to assess the safety and toxicity of different phytocomplex mixtures and their applications in the creation of nutraceuticals [4], pharmaceuticals or cosmetic preparations [80,81]. The only way for these strategies to be sustainable is to base them on the results of inter, multi and transdisciplinary professional and scientific research of networked research groups, which most often achieve the realisation of planned activities by devising new national and international projects.

### 2.3. AM Activity

AM peptides are natural plant defence agents contained in buckwheat seeds and bran, with AM powers against various pathogens [33]. Buckwheat AM peptides (Fa-AMP1, Fa-AMP2, BWI-1, BWI-2c, and FtTI) were detected, and their activity against fungi, G− and G+ bacteria was proven [27].

Zhong et al. [22], in their test, revealed that RUT, vitexin, and isovitexin exhibited weak inhibitory activity against all nine representative bacteria at the maximum concentration of 0.6 mg/mL, and that their MIC values should be higher than 0.6 mg/mL. Based on their results, QE could be the main antibacterial component of the crude methanolic extract (ME) of Tartary buckwheat germ cultures, which would find application as a potential antibacterial agent [22].

In the study of Alnour et al., the authors investigated the synergistic effects of RUT and QE with antibiotics gentamicin (an aminoglycoside) and ceftriaxone (a third-generation cephalosporin) against drug-resistant microorganisms [35]. Based on the different levels of resistance to AM agents and antibiotics, microorganisms were divided into three groups: multidrug-resistant (MDR), extensively drug-resistant (XDR), and pan drug-resistant (PDR). Clinical isolates included *Escherichia coli* (MDR), *Proteus mirabilis* (XDR), and *Klebsiella pneumoniae* (PDR). Rutin and quercetin restored the AM activity of the antibiotics against MDR and XDR strains, while no such effect was observed in the case of the PDR strain. QE demonstrated higher synergistic effects with ceftriaxone compared to RUT. Since RUT and QE are essentially present in human diets as constituents of fruits and vegetables, their use as nutraceuticals in adjuvant therapies in combination with antibiotics against drug resistance is a promising therapeutic strategy against superbug infections [35]. The thesis that is a pillar of toxicology can also be applied to dietary supplements: all compounds can be toxic if the dose is high enough, so attempts are made to use the lowest possible doses of various plant-based natural bioactive substances by preparing mixtures of phytocomplexes, whose synergy is proven and used. In the future, gaining in-depth knowledge of the efficacy and long-term safety of many dietary supplements will be imperative.

### 2.4. AO Activity of Buckwheat

Research on an in vitro human digestion model revealed that the AO activity of common buckwheat is increased by digestion in the small intestine via an increase in the BACs RUT and QE [82]. The AO capacity of QE from Tartary buckwheat was the strongest, compared with isoQE and RUT [83].

Based on the above, it can be concluded that the BACs found in buckwheat can be used in the pharmaceutical industry for the treatment of various health problems. Currently, the most attractive trend in the food industry is the creation of functional foods that offer health benefits. In relation to common buckwheat sprouts, ethanolic extracts of Tartary buckwheat sprouts have higher reducing power and free radical and superoxide anion removal activity, presumably due to the higher content of RUT and QE [40].

Other authors reported a very similar array of superior bioactivities of QE, as one (in buckwheat) of the most abundant, powerful and effective BACs from the plant kingdom [47].

Liu and Zhu ground whole seeds of Tatary buckwheat (cv “Jingkiao 2”) in a Brabender mill and separated small seeds using sieves (38GG and 7XXX) [84]. Particles that passed through the first sieve (<500 μm) were retained on the second (>200 μm) sieve. They were subsequently identified as crushed embryos, bran, aleurone and several hulls. They made up about 16% of the total weight of the grain, about 26% of husks, and about 58% of flour [84]. Oil and chlorophyll were removed from this material by refluxing with ether (80 °C, for 8 h), then extraction was performed with 61.57% ethanol solution, and the extract was washed with water to remove carbohydrates [84]. In this way, more than 95% of flavonoids were extracted, which alone represent about 47% of the mass of flavonoids in the whole seed. RUT is a proven dominant flavonoid. The authors suggested that such a procedure could be used to produce both flours and concentrates with high flavonoid content for potential incorporation into functional foods [84].

In their study, Wang et al. [79] reported on the phenolic content of Tartary buckwheat bran extract (TBBE). This bran was a by-product of flour produced from the “Kukiao 1” variety, grown in Sichuan Province, China. The bran was extracted three times with 95% ethanol at 55–65 °C. The weight of the dried TBBE was 9.85% of the weight of the extracted bran [79]. The concentrations of rutin and quercetin in TBBE were determined by the HPLC method, at the level of 8.96 and 83.84 mg/g, respectively [79]. After reflux hydrolysis (1 h at 80 °C) with HCl, the extract contained 19.47% QE [79]. The TPC in TBBE was measured by the Folin–Ciocalteu colorimetric test and was 158.44 μmol of gallic acid equivalents per gram [79].

In his interesting study, Liu [85] showed the proposed mechanisms by which phytochemicals in food can prevent cancer, stating that phytochemicals in common fruits and vegetables can have complementary and overlapping mechanisms of action (AO effects, elimination of free radicals, influence on gene expression in cell proliferation, cell differentiation, oncogenes, and tumour suppressor genes, apoptosis of cells, stimulation of enzyme activities in detoxification, oxidation, and reduction, immuno-stimulative effect, regulation of hormone metabolism, and antibacterial and antiviral effects. A significant recent review has focused on synergistic interactions of polyphenols and their effectiveness in the treatment of many diseases. The paper provides important information about the advantages of these compounds in combination. In addition to the synergistic effects of polyphenols, this review also described their specific health benefits and bioavailability in humans [86].

The effects of synergistic cocktails can be greater than the sum of the separate effects of individual BACs. At the same time, the presence of different species at the molecular level causes conflicting events that are useless from the point of view of improving health, which must also be considered [87]. Cell cultures are commonly used to evaluate the efficacy of BACs *in vitro* [88]. To avoid misinterpretations and inaccurate final results, it is necessary to use concentrations comparable to those detected in vivo, which can vary from nM to µM [89,90].

### 2.5. Buckwheat BAC Impact on Digestion of Carbohydrates (CHs) and Glucose (GLU) Absorption

Besides numerous BACs, buckwheat is rich in high-quality CHs, protein and amino acids, fatty acids, vitamins, and minerals. In comparison with other major grains, buckwheat has superior nutritional value [17,91].

The primary enzyme responsible for digesting CHs is intestinal α-glucosidase. When this enzyme is inhibited, the digestion process slows down, leading to reduced absorption of GLU from the gut into the bloodstream. By regulating blood GLU levels, dietary α-glucosidase inhibitors may help delay or prevent the onset of type II diabetes [92].

Qin et al. [92] studied the ability of Tartary buckwheat to inhibit α-glucosidase in (1) raw whole seeds; (2) whole seeds after soaking in water at 40 °C for 12–14 h; (3) whole seeds after steaming at 100 °C for 40–60 min; (4) whole seeds after drying at 150–200 °C for 5 min; (5) peeled semolina; and (6) Tartary buckwheat tea after roasting at 120–150 °C for 6–8 min, as shown in Table 5 [92].

Inhibition of a-glucosidase and a-amylase was shown to delay CH digestion and GLU absorption, and potentially reduce the risk of the onset of type II diabetes. Phenols (especially proanthocyanidins) inhibit the activity of the two enzymes mentioned above, so the high content of phenols in buckwheat increases its antidiabetic effect. When consuming bread enriched with buckwheat, a decrease in the glycaemic and insulin index was observed, probably due to the formation of indigestible starch after heating the buckwheat flour and the reduction of insulin resistance through D-chiro-inositol [67].

Lukšič et al. [93] compared the quality of sourdough bread made from common buckwheat flour (“Pira” variety) with that of Tartar buckwheat (an unnamed variety from Luxembourg). The researchers discovered that flour from Tartary buckwheat contained 1.47% RUT and 0.19% QE, while ordinary buckwheat flour did not contain any detectable amounts of these flavonoids [93]. They observed that the concentration of RUT decreased in Tartary buckwheat dough and continued to decrease during bread baking until it was undetectable in the final product. In contrast, the QE concentration was higher in the dough (0.82%), and then decreased to 0.51% after baking [93].

By determining the AOs measured in relation to the superoxide radical, the authors observed a different pattern of activity [93]. The activity of common buckwheat flour was two-thirds that of Tartary buckwheat flour. The AO activity of common buckwheat increased as the unfermented dough progressed through successive stages, while it decreased in the case of Tartary buckwheat. The activity of common buckwheat bread was half that of Tartary buckwheat bread, so it is clear that compounds other than RUT and QE contributed to the AO potential of buckwheat [93]. The authors also noted that both the crust and the crumb of Tartary buckwheat bread were more yellow and green in colour than regular buckwheat bread. Additionally, the cakes of the former were denser (1.1 g/cm^3^ vs. 0.9 g/cm^3^) [93].

Kočevar Glavač et al. [44] conducted a study on the concentrations of RUT and QE during the production of roasted Tartary buckwheat tea in South Korea. They took dry samples of raw seeds, whole seeds after steaming, seeds after steaming and hull removal, and kernels after roasting. These samples were ground, extracted with methanol, and then analyzed using HPLC. The raw grain contained 1.41% RUT and 0.004% QE, on a dry weight basis [44].

After steaming for 30 min, the amount of RUT decreased by almost half, while the amount of QE increased nearly six times. The dehulled seeds post-mating contained 0.826% rutin and 0.007% quercetin (QE) [44]. After roasting, different batches of the pulp contained between 0.327% and 6.28% RUT and between 0.039% and 0.082% QE while the large fragments of the steamed hulls contained 0.036% RUT and 0.040% QE [44]. Very fine hull fragments were much richer in RUT, at 1.61%, while containing less QE, at 0.022% [44].

## 3. Creation of Healthier and Functional Food Whose Ingredient Is Buckwheat

The results of numerous studies testify to the possible benefits for human health from the consumption of buckwheat in various forms (food, dietary supplements, home remedies or possibly pharmaceutical drugs) [3,18,32,42,55,58,71,72,73,74,75]. Counter-benefits must not be forgotten or excluded: adverse effects, especially due to contamination, must be considered [94].

Buckwheat is rich in BACs and can be processed into tea, beer and extruded products so that, in addition to bakery and gluten-free products, its application is as wide as possible. In food processing, extrusion technology is used to produce a large range of different quality products acceptable to consumers, such as pasta, modified flours, textural vegetable protein, meat analogues, snacks, and starch-based food [95].

Various Tartary buckwheat food products, such as alcoholic beverages, vinegar, tea, noodles, porridge, biscuits, cakes, and sprouts, have become commercially available in China [16]. Buckwheat bread is enjoyed by consumers across Europe due to its nutritional properties, AO capacity and the possibility of making gluten-free bread [96].

Tartary buckwheat has balanced amino acids and bioactive CH composition and a relatively high content of crude fibre, polyunsaturated fatty acids, and micronutrients compared with common buckwheat [97]. Buckwheat is rich in unsaturated fatty acids (74.5–79.3%), which have a beneficial effect on human health by preventing the occurrence of heart diseases, cancer, inflammation, and diabetes [58]. The AM activity assays of the methanol extract of Tartary buckwheat sprouts showed excellent activity toward most of the tested fungi and bacteria. QE presents the main BAC for contributing to AM activity [22]. Doctors and clinicians call buckwheat a “smart bioactive supplement”.

Buckwheat is the main pseudocereal with an excellent nutrition profile. It is rich in phytochemicals, vitamins and minerals. It represents a cheap and important source of protein because it contains more protein than cereals, and as such, it can alleviate malnutrition due to the deficiency of these nutrients in the diets of the population of developing countries. The pharmacological effects associated with bioactive peptides are AO and AM activities, cholesterol-lowering ability, hypoglycaemic effect, and antitumor activity [30]. BACs isolated from buckwheat can be used in the pharmaceutical industry for the treatment of various diseases.

Today, an attractive and growing trend in the food industry is the formulation of functional food that will have a proven positive effect on people’s health. Recently, buckwheat food products with good techno-functional and sensory properties are attracting consumers because they exhibit health benefits, especially in light of the fact that they are suitable for the diet of people with gluten intolerance. Further research and development efforts are needed to improve the sensory characteristics of gluten-free buckwheat products [3].

The subunits of polyphenols, (flavonoids, RUT, and QE), resulted in higher AO activity and 4.29 times higher total phenolic content (TPC) of enriched bread with buckwheat flour [42]. Different types/varieties of gluten-free buckwheat flour could be used as a substitute for wheat flour in a formulation of pasta and confectionery products (cookies) of up to 30%.

The AO potential of Tartary buckwheat is higher than that of common buckwheat. The loss of TPC from buckwheat-enriched whole wheat pasta (48.1–61.1%) indicates that pasta containing buckwheat flour may retain cooking quality [98].

Regardless of the higher TPC and AO activity, the unpleasant smell and bitter taste of pasta with buckwheat was especially pronounced if its share was greater than 10% [57].

Baking was reported to be a good approach for the accumulation of rutin in Tartary buckwheat flour, and thermally treated buckwheat flour can also be considered a source of rutin [45].

The development of ready-to-eat snacks made with up to 50% buckwheat, a gluten-free pseudocereal, associated or combined with other cereals or potatoes, would be very beneficial, as it could help obtain an acceptable product for celiacs and diabetics, fasting people, and other conventional consumers.

A buckwheat snack remained acceptable for six months [99]. Functional products could also be chocolate and chocolate pralines with buckwheat groats or flour. The salty vegan buckwheat spread and the sweet buckwheat and chocolate spread are examples of such products [7].

Bread enriched with buckwheat was found to have a higher content of dietary fibres and flavonoids, which reduced total cholesterol and LDL cholesterol in examined consumers. Bread made from 15% hulled buckwheat has an immunostimulant effect and contains higher amounts of insoluble β-glucan (when compared to wheat and unhulled bread) [42].

The uniqueness and exceptionality of this find was that the substance from the plant was obtained in the form of an individual substance—a crystalline powder, and not in the form of tincture, ointment, or extract, as is customary for use in phytopreparations. It has now become possible to use it in capsules or pills, in concentrations hundreds of times greater than those in former extracts. There was a revolution in the study of bioflavonoids when a natural BAC was obtained, as it could easily compete with synthetic drugs.

Today, the main raw material for obtaining DHQ on an industrial scale is Larch wood, which contains up to 2.5% flavonoids (DHQ accounts for 90–95% of the total flavonoid content). Many laboratory/clinical studies have shown that DHQ isolated from larch has high AO activity, usually significantly higher than in previously known natural analogues.

The current results suggest that the selected plant material and by-products could be attractive ingredients used to obtain various fortified clean-label products with potentially positive effects on human health and could be incorporated in manufacturing or industrial processes. The food waste was reduced, and a circular economic model was established, while simultaneously presenting environmental and economic advantages.

## 4. A Vision of Potential Future Research Using Buckwheat: Creating Healthier/Functional Meat Products

Meat is a nutritious food, rich in high-quality proteins, essential minerals, and B vitamins. However, it lacks antioxidants. From the need to preserve meat as an essential food, meat products were developed by combining different animal tissues with early preservation techniques (drying, salting, heating and smoking) and spices. Technological properties (processing yield, instrumental colour and texture) and oxidative and microbiological stability are in correlation with sensory characteristics. In general, all these properties depend on the ratio of protein, fat, and water, the use of non-meat ingredients (salt, nitrates, phosphates, etc.), preservation procedures and their interactions [100].

This is especially pronounced in ground meat products: (1) dry-fermented sausages —mostly smoked sausages dried in cold air and fermented (10–25 °C)—can be stored at ambient temperature for several months; (2) emulsified sausages (frankfurters, mortadella)—heat-treated, finely comminuted meat products—should be stored at 0–4 °C, usually up to 60 days; (3) fresh, minced meat products (burgers, patties, fresh sausages) which are intended for heat treatment before consumption should be stored at 0–2 °C, usually up to 7 days [100].

Suitable and safe food systems are part of the vision of the European Union (EU), which is described in the EU 2020 strategy. In October 2015, the WHO, through its International Agency for Research on Cancer (IARC), classified meat products as carcinogenic (Group I) and red meat as a potential carcinogen (Group IIa) based on research findings linking meat consumption to the development of colorectal and other types of cancer in both humans and animals [101,102,103,104].

Our interdisciplinary research group considers the project proposal that it designed to be founded and sustainable, and within it, our group plans complex activities to modify different groups of meat products by adding plant material or BACs from it, using their synergy.

Due to everything stated in the description of the proximate composition of buckwheat, the extremely high content of BACs and their numerous bioactivities and benefits for human health, we believe our consideration of the incorporation of different BACs from buckwheat into various meat products to be justified.

The addition of complex plant extracts (PEs) with AO effects to processed meat can lead to healthier products. Innovative preservation methods are necessary for both conventional and organic meat products to enhance sustainability and reduce the potential negative effects of processing and meat consumption. Complexes of plant extracts, containing high levels of specific phytochemicals derived from various plant materials and by-products, may have synergistic AM and AO effects. They could help prevent the growth of pathogens and bacteria that can cause spoilage in meat products, while also improving the quality, shelf-life, and safety of the meat [105,106,107,108,109,110].

BACs from natural (herbal and plant) sources exhibit different features, which can include nutritive and medicinal properties and AM and AO activities. Selected plants (we also selected buckwheat) contain different BACs with previously indicated properties, which extend the microbiological and oxidative stability of meat and plant-based meat analogues, as well as introduce BACs with medicinal properties (antimutagenic, anticancer, anti-inflammatory, etc.) [105,106,107,108,109,110].

Considering that the introduction of novel ingredients (especially as a replacement for conventional ones) could alter product properties, the first step will be to create model systems of meat and plant-based meat analogues. Model systems are a good way to start with this complex examination since this enables the examination of technological characteristics which are highly correlated with sensory properties. After the preparation of plant extracts (PEs) and lyophilizates (LYOs), model systems are designed in which potentially toxic additives (Na nitrite, Na ascorbate) are replaced by different amounts of PE or LYO. These model systems will be prepared according to the data presented in the literature [111] and our practical experience [112,113,114].

The following analyses will be conducted: proximate composition, measurement of pH and a_w_ values, technological properties [115,116], residual nitrite content [117], instrumental colour [118] and texture [119]. One-way and two-way ANOVA will be used to evaluate the results obtained in this first step. After a systematization of the results, according to the most promising formulations from model systems, typical meat products from the above groups will be prepared in the meat processing pilot plant.

Using the analysis mentioned in the first step, the prepared meat products will be examined after production and during the storage period appropriate for the type of products in question. Also, sensory analysis (numeric-descriptive scale with a nine-point system) and Check-All-That-Apply (CATA) method will be conducted [120]. Samples will be provided for food oral processing, to provide a more comprehensive insight into sensory properties [121].

Plant-based meat analogues are developed to mimic a real meat product characteristic, such as texture, appearance and mouthfeel, but are made entirely from plant materials. They can be pre-heated/cooked (e.g., pre-cooked burgers, nuggets and various sausages) or produced raw in a factory and later heated by the final consumer (e.g., raw burgers) [122]. The main ingredients used include plant lipids, proteins, polysaccharides (e.g., starches and fibres), and non-protein binding agents (e.g., methylcellulose and gums), flavour components and colouring ingredients [123]. The lipid amount in meat analogues matches that of their real meat counterparts, and they are used to enhance juiciness, flavour and overall mouthfeel of the final product [124]. Plant fats and oils used in meat analogues have a more favourable fatty acid profile (contain a greater portion of polyunsaturated fatty acids) and do not contain cholesterol, unlike animal-derived products. Highly unsaturated oils are more prone to oxidative changes during storage, which can have a huge effect on stability, shelf-life and sensory quality of the final product [123]. Two types of products will be made: (1) pre-cooked sausages and (2) raw burgers. Sausages will be made with a combination of protein isolate, vegetable oil, hydrocolloids and spices, emulsified in a bowl chopper, filled into casings, steamed in the steam-oven and stored. Raw burgers will be made by mixing previously hydrated texturized vegetable protein (TVP) with vegetable oil, methylcellulose (as binder) and spices in a Hobart mixer; afterwards, patties will be shaped and stored in chilled conditions. In both groups, additional products will be made with an addition of natural BACs from plant sources and their blends.

## 5. Conclusions

Research into the application potential of buckwheat should be directed towards the creation of added-value products (functional food, nutraceuticals and products for industrial use) that would commercialize its use and open new market segments.

If we decide to market the nutritionally valuable ingredients of buckwheat and its bioactive ingredients to consumers through food, the aim of the research should be improved quality of existing foods and the creation of new, healthier or functional products with desirable techno-functional properties, enriched with the highest level of BACs (in accordance with WHO directives and national legislation).

The aim of the combination of different plant products is to achieve synergistic antimicrobial and health-improving effects and the ability to lower the concentration of each component to ensure a lower effect on the sensory properties of modified/conceptual innovative foods of animal and plant origin. Modifying food in the above way is “clean label” and consumer-friendly.

To date, there are no studies that report complete agreement on unsurpassed reference models containing the definition of combined effects and synergies between bioactive ingredients of plant origin, although there are models used in herbal drug research and the design of natural antioxidants and food preservatives. In the future, it will be necessary to create such models of desirable properties with wide applications in the food industry, phytocosmetology, as well as medicine.

## Figures and Tables

**Table 1:** Nutritional composition of buckwheat [3].

Compounds (%)	Average	Cultivars	
		Common buckwheat	Tartary buckwheat
Protein	13.07	12.30	13.15
Carbohydrates (CH)	56.00	5450	57.40
Lipid	2.52	3.80	3.84
Dietary fibre	11.94	7.00	10.60
Ash	1.67	2.00	2.70
Other compounds (soluble CH, phenolic compounds, organic acid, nucleotides)	14.80	18.40	10.53

Adopted from Sofi et al., 2022 [3].

**Table 2:** Buckwheat (*Fagopirum spp.*) crop production at world and national levels [25].

Country	Year	Area Harvested (ha)	Yield (t/ha)	Production (t)
World		2,088,527	1.16	2,268,191
Russia	2020	821,366	1.09	892,160
China, mainland	2020	624,780	0.81	503,988
France	2017	74,883	3.52	263,485
Poland	2027	78,027	1.45	113,113
Ukraine	2020	84,100	1.16	97,640
USA	2020	81,620	1.06	86,397
Brazil	2020	46,416	1.40	65,117
Lithuania	2017	48,499	1.10	53,221
Japan	2020	66,600	0.67	44,800
Kazakhstan	2020	55,076	0.73	40,094
Belarus	2020	27,354	1.03	28,300
United Republic of Tanzania	2020	24,295	1.06	25,772
Latvia	2017	18,300	0.93	17,100
Nepal	2020	10,369	1.13	11,724
Canada	2020	9800	0.91	8900
Estonia	2017	5278	0.64	3385
Slovenia	2017	3647	0.80	2909
Bhutan	2020	2004	1.35	2701
Czech Republic	2017	887	2.55	2262
Bosnia and Herzegovina	2020	833	1.56	1301
Korea	2020	1600	0.97	1549
Hungary	2017	969	0.94	909
Croatia	2017	695	0.90	624
Slovakia	2017	429	0.86	367
South Africa	2020	579	0.40	234
Georgia	2020	106	1.11	118
Kyrgyzstan	2020	10	1.70	17
Republic of Moldova	2020	5	0.80	4

Adopted from Kreft et al., 2022 [25].

**Table 3:** Bioactivities and BACs of buckwheat.

Bioactivity	Bioactive Compounds (BACs)		References
Antimicrobial	Antimicrobial peptides	fa-AMP1, fa-AMP2, FtTI	[3,22,26,27,28,29,30,31,32,33,34,35,36]
	Flavonoids	Rutin, quercetin, kaemferol, isoorientin	
	Phenolic acids	Caffeic acid	
Antibacterial and antiviral	Phenolic acids	Chlorogenic acid, hydroxybenzoic acid	[17,37,38,39]
	Flavonoids	Epicatechin, luteolin, kaempferol, quercetin, rutin	
Antioxidant	Flavonoids	Rutin, orientin, vitexin, quercetin, isovitexin, isoorientin, catechin	[3,18,19,20,21,22,23,24,25,32,40,41,42,43,44,45,46,47,48,49,50,51,52,53,54,55,56,57]
	Stilbenes	Resveratrol	
	Phenolic acids	*p*-Hydroxybenzoic, ferulic, protocatechuic, *p*-coumaric, gallic, caffeic, vanillic, chlorogenic, syringic, and salicylic acids	
Cardioprotective	Flavonoids	Rutin, quercetin, apigenin, isorhamnetin, kaempferol, luteolin, naringenin	[3,17,22,23,32,55,58]
	Phenolic acids	Ferulic acid, gallic acid, cinnamic acid, syringic acid	
Antithrombotic	Flavonoids	Rutin, kaempferol, myricetin, quercetin	[3,17]
Anticancer (mammary, colon, skin, and other cancers)	Flavonoids	Rutin, quercetin	[15,18,22,23,32,49,55], [58,59,60,61,62,63,64,65]
	Phenolic acids	Ferulic acid, gallic acid	
Antiulcer	Flavonoids	Rutin, kaempferol, quercetin	[17]
Hepatoprotective	Flavonoids	Rutin, quercetin	[3,17,66]
Antidiabetic	Flavonoids	Proanthocyanidins, rutin, quercetin	[3,18,32,58,67,68,69]
Anti-inflammatory and Antifatigue effects	Carbohydrates	Polysaccharides	[3,22,32,58,70]
	Flavonoids	Apigenin, isoorientin, isovitexin, chrysin, hispidulin, hesperidin, luteolin, rutin, quercetin	
Antihyperlipidemia	Carbohydrates	Polysaccharides	[3,32]
	Flavonoids	Quercetin	
Cholesterol-lowering	Carbohydrates	Fagopyritol A1	[18,32,42,71,72]
	Flavonoids	Rutin, quercetin	
Antihypertensive	Flavonoids	Quercetin	[3,18,32]
Antineurodegenerative	Flavonoids	Galangin, kaempferol, myricetin, rutin, quercetin	[3,14,23,32,73]
Antigenotoxicity	Flavonoids	Rutin, quercetin	[3,74]
Cognition-improving, and mental health diseases	Flavonoids	Quercetin	[18,32,75]
	Phenolic acids	Chlorogenic acid	
Anti-ageing	Phenolic acids	Caffeic acid	[55]
Immunostimulating	Carbohydrates	Insoluble β-glucan	[42]

**Table 4:** Total phenolics and in vitro antioxidant activity of selected gluten-free flours from cereals, pseudo-cereals and legumes [76].

Gluten-Free Flour	Total Phenolics (mg GAE 100 g^−1^)	In vitro Antioxidant Capacity	
		FRAP (μmol GAE 100 g^−1^)	ORAC (μmol TE 100 g^−1^)
Buckwheat	275.5 ± 2.7	494.2 ± 35.9	8177.1 ± 68.4
Black chickpea	180.2 ± 0.7	396.2 ± 3.8	1710.7 ± 18.9
Amaranth	57.0 ± 1.0	76.2 ± 3.8	1360.4 ± 28.5
Kidney bean	95.5 ± 3.6	198.1 ± 7.5	3460.4 ± 61.7
Adzuki bean	124.6 ± 1.4	330.9 ± 3.7	2751.1 ± 108.1
Black bean	90.3 ± 5.9	250.4 ± 7.5	4033.7 ± 109.9
Red lentil	71.9 ± 1.5	106.7 ± 3.8	1706.7 ± 24.7
Red quinoa	130.2 ± 2.5	230.8 ± 7.5	2752.6 ± 54.4
Black quinoa	130.2 ± 1.9	289.6 ± 7.5	3236.3 ± 52.7
Orange rice	75.9 ± 0.7	256.9 ± 26.4	2550.7 ± 79.6
Ermes rice	68.4 ± 1.6	200.3 ± 7.5	1443.7 ± 8.9
Wild rice	81.9 ± 1.4	195.9 ± 11.3	3181.5 ± 35.7
Red sorghum	108.4 ± 5.1	324.4 ± 9.9	4734.8 ± 59.5
White sorghum	52.3 ± 0.2	145.9 ± 7.5	2236.3 ± 63.1

The data in the table are taken from Rocchetti et al., 2019 [76].

**Table 5:** Concentrations (mg/g dry weight) of extractable total phenolic content (TPC), DPPH radical-scavenging activity (μmol TE/g dry weight) and α-glucosidase inhibitory activity (%) in collected Tartary buckwheat [92].

Tartary Buckwheat	Extractable TPC	DPPH Radical Scavenging Activity	α-Glucosidase Inhibitory Activity (%)
Raw whole seeds	12.992 ± 0.08	69.214 ± 1.75	28.978 ± 4.72
Soaked whole seeds	16.224 ± 0.34	92.987 ± 3.15	37.875 ± 3.68
Steamed whole seeds	10.539 ± 0.12	45.849 ± 4.25	22.551 ± 4.98
Dried whole seeds	8.752 ± 0.18	36.218 ± 3.28	19.986 ± 4.85
Dehulled groats	5.412 ± 0.23	21.896 ± 2.72	15.875 ± 2.36
Tartary buckwheat tea	4.751 ± 0.25	19.286 ± 2.92	15.567 ± 2.68

The data in the table are taken from Qin et al., 2013 [92].

## Data Availability

No new data were created or analyzed in this study. Data sharing is not applicable to this article.
